# In Situ Monitoring of the Antibacterial Activity of a Copper–Silver Alloy Using Confocal Laser Scanning Microscopy and pH Microsensors

**DOI:** 10.1002/gch2.201900044

**Published:** 2019-07-15

**Authors:** Nicole Ciacotich, Kasper Nørskov Kragh, Mads Lichtenberg, Jens Edward Tesdorpf, Thomas Bjarnsholt, Lone Gram

**Affiliations:** ^1^ Elplatek A/S Bybjergvej 7 DK‐3060 Espergærde Denmark; ^2^ Department of Biotechnology and Biomedicine Technical University of Denmark Søltofts Plads Bldg. 221 DK‐2800 Kgs Lyngby Denmark; ^3^ Department of Immunology and Microbiology Costerton Biofilm Center Faculty of Health and Medical Sciences University of Copenhagen Blegdamsvej 3B DK‐2200 Copenhagen N Denmark; ^4^ Department of Clinical Microbiology Rigshospitalet Juliane Maries vej 22 2100 Copenhagen Ø Denmark

**Keywords:** antibacterial activity, bacterial biofilms, confocal laser scanning microscopy, copper–silver alloy, electroplating

## Abstract

The antibacterial efficacy of a copper–silver alloy coating under conditions resembling build up of dry surface bacterial biofilms is successfully demonstrated according to US EPA test methods with a ≥99.9% reduction of test organisms over a 24 h period. A tailor‐made confocal imaging protocol is designed to visualize in situ the killing of bacterial biofilms at the copper–silver alloy surface and monitor the kinetics for 100 min. The copper–silver alloy coating eradicates a biofilm of Gram‐positive bacteria within 5 min while a biofilm of Gram‐negative bacteria are killed more slowly. In situ pH monitoring indicates a 2‐log units increase at the interface between the metallic surface and bacterial biofilm; however, the viability of the bacteria is not directly affected by this raise (pH 8.0–9.5) when tested in buffer. The OH^−^ production, as a result of the interaction between the electrochemically active surface and the bacterial biofilm under environmental conditions, is thus one aspect of the contact‐mediated killing of the copper–silver alloy coating and not the direct cause of the observed antibacterial efficacy. The combination of oxidation of bacterial cells, release of copper ions, and local pH raise characterizes the antibacterial activity of the copper–silver alloy‐coated dry surface.

## 1 Introduction

Microorganisms attach to both inert and biological surfaces and readily form biofilms.^1^ This is especially problematic in healthcare settings, where dry surface biofilms can survive for extended periods on a multitude of surfaces.^1^, ^2^, ^3^ Microbial communities assembled in a biofilm are less susceptible to biocides, antibiotics, and physical stress.^1^ Therefore, dry surface biofilms can play a significant role in transmission of healthcare‐associated infections, and dry environmental surfaces are a persistent source for the transfer of pathogens.^1^, ^3^

Copper and copper alloy surfaces have been receiving increasing attention in the recent years, as a method for reducing such bacterial attachment and biofilms and subsequently the spread of pathogenic microorganisms in healthcare settings, thus potentially alleviating the occurrence of hospital mediated infections.^4^, ^5^, ^6^ Evidence of their antibacterial properties from laboratory experiments has led to several field test studies in healthcare facilities in Europe and USA to validate their performances in real‐life conditions.^5^, ^7^, ^8^ In 2015, the United States Environmental Protection Agency (US EPA) released tailored protocols for testing and evaluating the antibacterial efficacy of copper and copper alloy surfaces with the intention of providing harmonized test conditions closely resembling real‐life applications of such surfaces, e.g., environmental indoor items in healthcare facilities.^9^, ^10^, ^11^ The first two protocols allow evaluation of the sanitizing efficacy of copper alloys on test organisms after 2 h exposure and after a prolonged exposure to a bacterial contamination accumulated over a 24 h interval.^9^, ^10^ Several copper‐based surfaces have demonstrated antimicrobial effectiveness according to these protocols.^12^, ^13^

A copper–silver (90–10 wt%) alloy laser‐clad coating for stainless steel exhibited enhanced killing of *Escherichia coli*, as compared to the pure elements, and it was correlated with an 28‐fold increased release of copper ions.^14^ Similarly, a copper–silver (60–40 wt%) alloy electroplated coating has recently demonstrated strong antibacterial activity against *Staphylococcus aureus* and *E.coli* when tested in suspension.^15^, ^16^ In these test conditions, the copper–silver alloy‐coated surfaces released copper ions in the bacterial suspension and the release was enhanced by a concentrated bacterial suspension or presence of nutrient broth.^15^ Copper, the less noble alloying element, protected silver from dissolution by its preferential oxidation, according to the principle of galvanic corrosion.^15^, ^16^ This was confirmed by measurements of silver that was detected only as traces in the suspensions.^14^, ^16^ Therefore, the galvanic coupling of the two metals in the alloy coating induces oxidation of copper, resulting in release of copper ions, and reduction reaction on silver, leading to a local pH increase, under environmental conditions, e.g., in the presence of chlorides.^15^, ^16^ When bacteria are exposed to a copper–silver alloy‐coated surface, a galvanic series is established, where silver holds the highest electrochemical potential followed by copper and bacteria.^16^, ^17^

It is currently understood that bacteria are killed on dry copper surfaces through a contact‐mediated killing process.^18^ Copper dissolving from the surfaces and accumulating at the aqueous interface between the metallic substrate and bacterial cells causes severe membrane damage and overload of copper ions in the cytoplasm.^18^, ^19^ This scenario is quite different from killing of bacteria by copper ions in suspension or in culture, where the “free” copper ions concentration is lower by several orders of magnitude and bacteria are under growth conditions.^18^

The antibacterial efficacy of the newly developed copper–silver alloy against bacteria in suspension has been evaluated as mentioned, and this could resemble exposure to disinfectants, detergents, and hand sweat in the intended applications.^16^ However, such surfaces will mostly face dry or humid conditions in a healthcare setting. It is possible that the antibacterial efficacy of this alloy would be enhanced in this dry scenario, also considering that the copper–silver alloy is an electrochemical active surface and is expected to have a different behavior than other copper alloy surfaces.^15^ The surface contact is the well‐established primary killing factor of copper alloys surfaces and the killing rate is crucial for any real‐life application. Moreover, evidence suggested that the killing process initiates immediately after surface contact is established, and the exposed surface area and rate of release of copper ions can easily influence the overall rate of contact killing.^20^, ^21^ Therefore, the aim of this study is to determine the antibacterial activity of the copper–silver alloy coating under closer to real‐life conditions, e.g., under dry conditions allowing a bacterial biofilm build‐up.

## 2 Results and Discussion

### 2.1 Validation of Antibacterial Efficacy Through US EPA Test Methods

Test cultures, neutralizer solution and carriers successfully passed all the sterility, viability, quantitation and antimicrobial susceptibility controls carried out following the guidelines of the US EPA Test methods procedures.^9^, ^10^ The initial concentration of test organisms was ≈10^8^ CFU mL^−1^ (**Table** **1**) in line with the US EPA Test methods for Efficacy as Sanitizer (Protocol 1) and Continuous Reduction of Bacterial Contamination (Protocol 2) of Copper Alloy Surfaces.^9^, ^10^ *Staphylococcus aureus* ATCC 6538 and *Staphylococcus aureus* MRSA ATCC 33592 were effectively inactivated by the copper–silver alloy coating with a 5‐log reduction compared to the stainless steel control carriers after 2 h (Protocol 1) and at all time points over the 24 h time interval (Protocol 2), yielding a percent reduction greater than 99.9% (Table 1).^9^ Copper–silver alloy‐coated surfaces also reduced *Enterobacter aerogenes* ATCC 13048 levels with 5‐logs compared to the stainless steel control carriers in Protocols 1 and 2 at all time points except after 2 h, where the level on stainless steel controls was ≈10^2^ CFU per carrier. However, the percent reduction of the copper–silver alloy–coated compared to uncoated stainless steel surfaces was greater than 99.9% both in Protocols 1 and 2 (Table 1).^9^ In Protocol 1 *Pseudomonas aeruginosa* ATCC 15442 was able to survive on copper–silver alloy‐coated surfaces to a geometric mean of 5.9 CFU per carrier, therefore the percent reduction was 99.9% compared to the stainless steel control surfaces, where the geometric mean of surviving *P. aeruginosa* was 1.1 × 10^4^ CFU per carrier. However, the percent reduction was greater than 99.9% in Protocol 2 from 2‐log reduction (after 2 h) to 4‐log reduction (after 6, 12, 18, 24 h) (Table 1). Therefore, the copper–silver alloy‐coated surfaces passed successfully the acceptance criteria of the test methods, i.e., a percentage reduction ≥99.9% after 2 h exposure and ≥99.0% at all‐time points over the 24 h time interval, respectively.^9^, ^10^

**Table 1:** Results of US EPA test methods for efficacy as sanitizer (Protocol 1) and continuous reduction of bacterial contamination (Protocol 2) of copper–silver alloy‐coated surfaces. Limit of detection (LOD) = 2.3 CFU per carrier

Microorganism	Protocol	Inoculum CFU mL^−1^	Recovered geometric mean numbers CFU per carrier		Percentage reduction
			Control	Test	
*S. aureus* ATCC 6538	1	1.7 × 10^8^	2.6 × 10^5^	<LOD	>99.9%
	2–2 h	2.7 × 10^8^	9.9 × 10^4^	<LOD	>99.9%
	2–6 h		4.0 × 10^4^	<LOD	>99.9%
	2–12 h		9.3 × 10^4^	<LOD	>99.9%
	2–18 h		1.1 × 10^5^	<LOD	>99.9%
	2–24 h		9.0 × 10^4^	<LOD	>99.9%
*E. aerogenes* ATCC 13048	1	8.7 × 10^8^	8.0 × 10^5^	2.4	>99.9%
	2–2 h	5.6 × 10^8^	1.3 × 10^2^	<LOD	>99.9%
	2–6 h		1.6 × 10^5^	<LOD	>99.9%
	2–12 h		1.8 × 10^5^	<LOD	>99.9%
	2–18 h		7.5 × 10^4^	<LOD	>99.9%
	2–24 h		2.4 × 10^4^	<LOD	>99.9%
*P. aeruginosa* ATCC 15442	1	7.0 × 10^8^	1.1 × 10^4^	5.9	99.9%
	2–2 h	9.0 × 10^8^	2.4 × 10^2^	<LOD	>99.9%
	2–6 h		2.3 × 10^4^	<LOD	>99.9%
	2–12 h		1.2 × 10^4^	<LOD	>99.9%
	2–18 h		3.8 × 10^4^	<LOD	>99.9%
	2–24 h		2.3 × 10^4^	<LOD	>99.9%
MRSA ATCC 33592	1	3.3 × 10^8^	1.0 × 10^6^	<LOD	>99.9%
	2–2 h	1.7 × 10^8^	8.9 × 10^4^	<LOD	>99.9%
	2–6 h		1.0 × 10^5^	<LOD	>99.9%
	2–12 h		1.2 × 10^5^	<LOD	>99.9%
	2–18 h		1.9 × 10^5^	<LOD	>99.9%
	2–24 h		1.4 × 10^5^	<LOD	>99.9%

### 2.2 Confocal Laser Scanning Microscopy (CLSM) and Biomass Quantification

In order to visualize bacterial cells with a compromised membrane after exposure to copper surfaces, live/dead staining technique and fluorescence microscopy are the obvious choices that easily allow differentiation between bacterial cells with intact (green fluorescence) and compromised (red fluorescence) membranes. However, it was observed that regular fluorescence indicator dyes lose their fluorescence upon contact with metallic copper surfaces, due to the light absorption of copper.^18^, ^20^ Cells could be simply removed from surfaces prior to the staining procedure and then inspected, but this would only allow a post‐visualizaton of the damaging effect caused by contact killing after set exposure times and not an in situ follow‐up at the copper surfaces.^18^

**Figure 1:**
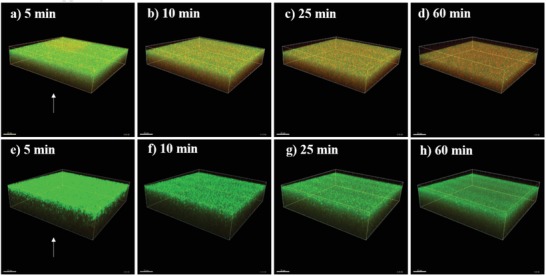
*Pseudomonas aeruginosa* PAO1 live and dead cells exposed to a–d) copper–silver alloy‐coated and e–h) uncoated AISI 316 surfaces monitored at the beginning of a,e) the exposure, and after b,f) 10 min, c,g) 25 min and d,h) 60 min. The arrow indicates the position of the metallic surfaces. Cells are stained with a modified live/dead dye stain mixture (0.2% of SYTO 9 green‐fluorescent nucleic acid and 0.2% of SYTOX AADvanced dead cell stain) and live cells appear green and dead cells stain red.

**Figure 2:**
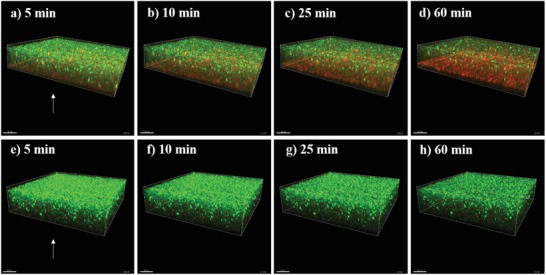
*Pseudomonas aeruginosa* PAO1 live and dead cells exposed to a–d) copper–silver alloy‐coated and e–h) uncoated AISI 316 surfaces monitored at the beginning of a,e) the exposure, after b,f) 10 min, c,g) 25 min and d,h) 60 min. The arrow indicates the position of the metallic surfaces. Cells are stained with a modified live/dead dye stain mixture (0.2% of SYTO 9 green‐fluorescent nucleic acid and 0.2% of SYTOX AADvanced dead cell stain) and live cells appear green and dead cells stain red.

**Figure 3:**
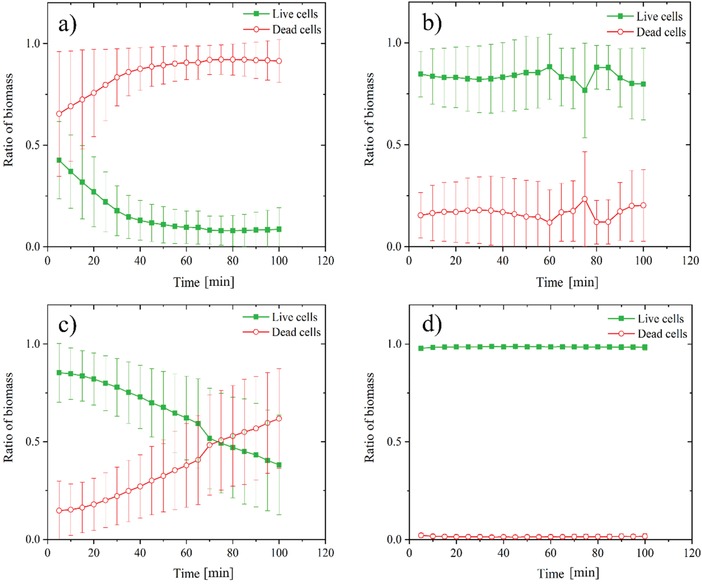
Ratio of *Staphylococcus aureus* 8325 live and dead cells exposed to a) copper–silver alloy‐coated and b) uncoated AISI 316 surfaces and *Pseudomonas aeruginosa* PAO1 live and dead cells exposed to c) copper–silver alloy‐coated and d) uncoated AISI 316 surfaces.

Here, *S. aureus* 8325 (**Figure** **1**) and *P. aeruginosa* PAO1 (**Figure** **2**) cells were exposed and visualized directly at the surface of copper–silver alloy‐coated and uncoated AISI 316 samples using a modified live/dead staining procedure and CLSM during a time interval of 100 min. Within the first 10 min of exposure to the copper–silver alloy‐coated surfaces, the number of *S. aureus* 8325 dead cells (red) surpassed the number of live cells (green) (**Figures** 1 and **3**). After 25 min, the remaining live cells were less than 20% (Figure 3a) and the majority of cells appeared red after 60 min (Figure 1d). In contrast, *S. aureus* 8325 cells exposed to AISI 316 surfaces remained alive (Figure 1e–h) and their percentage was approximately above 80% over the whole exposure period (Figure 3b). The number of *P. aeruginosa* PAO1 live cells exposed to copper–silver alloy‐coated surfaces decreased over time from the beginning of exposure up to 60 min (Figure 2a–d), when the ratio of live and dead cells shifted in favor of the latter and the number of dead cells started to increase (Figure 3c). On AISI 316 surfaces, *P. aeruginosa* PAO1 cells remained alive (Figure 2e–h) and their percentage was close to 100% (Figure 3d) over the 100 min exposure period.

The direct visualization at the metal surface confirmed the antibacterial efficacy of copper–silver alloy‐coated surfaces as compared to uncoated stainless steel controls, as also observed in the US EPA protocols testing. The copper–silver alloy‐coated surfaces caused a more rapid killing of *S. aureus* than of *P. aeruginosa* and a lower percentage reduction in numbers of *P. aeruginosa* was also observed in the US EPA Test Method for Efficacy as Sanitizer (Protocol 1). Copper oxide impregnated non‐porous solid surfaces were tested using the US EPA test protocols and did not reach a 99.9% reduction of *P. aeruginosa* in all tests.^13^ Thus, these findings might suggest that *P. aeruginosa* can, to some extend, withstand exposure and contact to copper‐based surfaces. During contact killing, when copper dissolves from the copper–silver alloy‐coated surface as triggered by the presence of the bacterial film, copper ions accumulate in that limited space.^18^ Membrane damage then occurs and copper ions enter the bacterial cytoplasm.^18^, ^19^ The presence of two cell membranes separated by a periplasmic space in Gram‐negative bacteria and potentially the different mechanisms of copper homeostasis, active before contact killing inhibits the metabolic activities, can explain the observed delay in killing of *P. aeruginosa*. However, copper homeostasis mechanisms in Gram‐positive and negative bacteria have not yet been fully unraveled and detecting the concentration of free copper ions at the interface poses serious practical issues.^18^

### 2.3 pH Monitoring at Copper–Silver Alloy‐Coated and Uncoated Surfaces

**Figure 4:**
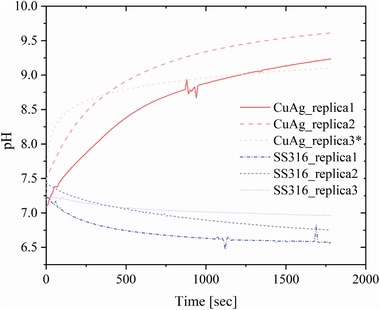
pH monitoring at the copper–silver alloy‐coated and uncoated SS316 surfaces with 0.15 m NaCl 0.5% agarose matrix loaded with *Staphylococcus aureus* 8325 suspension. *the replicate was fitted with a model (indicated in the experimental section) that allowed extrapolation of its initial pH rise, due to a slower positioning of the sensor.

**Figure 5:**
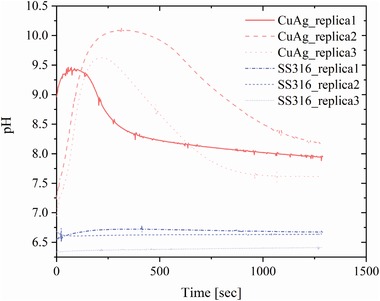
pH monitoring at the copper–silver alloy‐coated and uncoated SS316 surfaces with unloaded 0.15 m NaCl 0.5% agarose matrix.

Close to the interface between the layer of *S. aureus* 8325 suspension and the copper–silver alloy‐coated surface, the pH increased with a rate of ≈0.14 pH units min^−1^ reaching a plateau at pH above 9.0 after 20 min (**Figure** **4**). In contrast, pH at the interface between the layer of *S. aureus* 8325 suspension and the AISI 316 surface decreased after 10 min from values between 7.5 and 7.2 to values between 7.1 and 6.7 with a rate of ≈0.03 pH units min^−1^. After 20 min, the pH reached plateau values between 7.0 and 6.5 (Figure 4). This clearly demonstrates the electrochemical activity of the copper–silver alloy‐coated surface and the occurrence of the reduction reaction (O_2_ + 2H_2_O + 4*e* ^−^ → 4OH^−^) at the aqueous interface with production of OH^−^ ions that raised locally the pH. If the copper–silver alloy is immersed in chloride‐containing environments, galvanic corrosion conditions are established. In a 0.15 m saline solution, silver and copper exhibit corrosion potentials of 120 and 15 mV (vs standard hydrogen electrode), respectively.^16^ Therefore, silver, the nobler metal in the galvanic couple, is protected at the expenses of copper and its dissolution rate increases with the increasing silver content in the alloy.^22^ The membrane potential of *S. aureus* in the pH range from 5.0 to 7.0 is in the order of ‐100 mV (measured as distribution of [^3^H]tetraphenylphosphonium TPP+).^17^ Thus, in a three‐element system consisting of the two alloyed metals and the *S. aureus* 8325 suspension in presence of the 0.15 m NaCl agarose matrix, a galvanic series is also established, where silver holds the highest electrochemical potential followed by copper and the bacterial material.^16^, ^17^ Consequently, the organic material readily oxidizes, since it possesses the lowest electrochemical potential, whereas the metallic alloy results the site of the reduction reaction. Copper is well known for its catalytic activity,^23^, ^24^, ^25^ and this had potentially influenced the reaction rate and so the OH^−^ production rate. Moreover, the presence of the bacterial biofilm prevented the formation of copper oxide, maintaining the alloy‐coated surface active, and provided enough material for the redox reaction to proceed at an equilibrium rate as indicated by the plateau after 20 min (Figure 4). In contrast, uncoated stainless steel was simply an inert substrate and the pH reduction was probably the result of an adjustment to optimal pH conditions from the unchallenged bacterial suspension in contact with the 0.15 m NaCl agarose matrix. If *S. aureus* 8325 suspension was not present at the interface between the agarose matrix and the copper–silver alloy‐coated surfaces, pH increased with a rate of 0.69 pH units min^‐1^ from values between 7.0 and 7.5 (**Figure** **5**, replicas 2 and 3) to peak values between 9.5 and 10.0 after 4 min. In the case of replica 1 (Figure 5), the pH was already 9.0 at the beginning of the measurements and it reached its peak value of 9.4 after 1 min. Then, pH started to decrease with a slower rate of ≈0.12 pH units min^‐1^ to reach values between 8.2 and 7.6 after 20 min. When the pH was monitored at the interface between the agarose matrix and the AISI 316 surfaces, it maintained approximately constant values between 6.4 and 6.7 for the whole duration of the measurement (Figure 5).

Once the contact between the 0.15 m NaCl agarose matrix and the copper–silver alloy‐coated surface was established, the redox reaction readily initiated. In these conditions of bimetallic corrosion, the reduction reaction at silver sites produced OH^−^ that raised the pH and simultaneously copper dissolved from the alloy‐coated surface. Copper ions subsequently reacted with the surrounding environment forming copper oxide Cu_2_O.^26^ The presence of Cu_2_O led then to a pH decrease because of the establishment of new equilibrium conditions at the metal surface. Stainless steel surfaces were an electrochemically inactive substrate also in absence of a bacterial suspension layer, as clearly indicated by the constant pH value measured at the interface.

### 2.4 Exposure of *S. aureus* 8325 to 1 m Tris–HCl Buffer at pH 7.0 to 9.5

Due to the observed changes in pH at the copper–silver alloy‐coated surface, we questioned whether this increase in pH was the main cause of the rapid contact killing. Therefore, we exposed *S. aureus* 8325 at an initial concentration of ≈10^9^ CFU mL^−1^ (corresponding to OD_600_ 2.0) to pH 8.0, 8.5, 9.0, and 9.5 in 1 m Tris–HCl buffer and incubated it at 25 °C for 1 and 24 h.

After 1 h exposure, *S. aureus* survived at levels between 10^8^ and 10^9^ CFU mL^−1^ and the cell level remained above 10^7^ CFU mL^−1^ also after 24 h (**Table** **2**). There was no statistically significant difference (*P* > 0.01) in survival after 1 and 24 h at the different pH. The 1‐log reduction after 24 h exposure was caused by the buffering conditions, and it is not comparable to the 4 to 5‐log reduction by the contact killing observed in the US EPA tests after 2 h. This indicates that *S. aureus* survival was not significantly influenced by the exposure to 1 m Tris–HCl buffer at 8.0–9.5 pH range. Therefore, it is not likely that the increase in pH at the copper–silver alloy‐coated surface is the major cause of bacterial reduction, and it would rather have a secondary role in the contact‐mediated killing by the copper–silver alloy‐coated surface. Under environmental conditions and in presence of a bacterial biofilm at the interface, the galvanic coupling of copper and silver in the alloy would induce a redox reaction. Bacterial cells in contact with the alloy would oxidize, as they hold the lowest potential and a reduction reaction, resulting in OH^−^ production and local pH raise, would occur at the metal sites.

**Table 2:** *Staphylococcus aureus* 8325 survival after 1 h and 24 h exposed to 1 m Tris–HCl buffer at pH 8.0, 8.5, 9.0, 9.5

pH	Average Log CFU mL^‐1^ after 1 h	Average Log CFU mL^‐1^ after 24 h
8.0	8.3 ± 1.6	7.9 ± 1.3
8.5	8.9 ± 0.5	7.2 ± 0.6
9.0	9.2 ± 0.5	8.7 ± 0.5
9.5	9.4 ± 0.2	7.8 ± 0.5

## 3 Conclusion

In this study, we demonstrated the antibacterial properties of a copper–silver alloy coating against bacterial contamination under dry and real‐life like conditions. We used the US EPA test methods for efficacy as sanitizer and continuous reduction of bacterial contamination and a direct visualization by CLSM. The alloy passed successfully the EPA acceptance criteria of both test methods with a percentage reduction equal (*P. aeruginosa* ATCC 15442) or greater than 99.9% after 2 h exposure, and greater than 99.9% at all‐time points over the 24 h time interval.

During the in situ monitoring of the contact killing at copper–silver alloy‐coated and uncoated surfaces, we found a higher killing rate against bacterial biofilm of *S. aureus* 8325 than *P. aeruginosa* PAO1. Gram‐positive alive cells were markedly reduced within the first minutes of exposure, whereas the ratio between alive and dead Gram‐negative cells shifted toward the latter after 60 min of exposure. Membrane differences and different mechanisms of copper homeostasis may explain the slower killing rate in case of *P. aeruginosa* PAO1 bacterial biofilm.

pH measurement and monitoring at the copper–silver alloy‐coated surfaces revealed a fast increase and reaching a plateau at pH 9.0 after 20 min, when *S. aureus* 8325 suspension was present at the interface between the surface and the agarose saline matrix. In absence of bacterial material, pH rapidly increased to ≈9.5 and dropped due to the formation of Cu_2_O. No pH increase was detected at the uncoated control AISI 316 surface, due to the lack of electrochemical activity. When *S. aureus* 8325 was suspended in buffer solutions at different pH (range 8.0–9.5) no significant reduction in numbers was observed, indicating that pH could not be the sole responsible of the observed antibacterial properties. Therefore, OH^−^ production is probably not the main reason for the contact‐mediated killing phenomenon. Under environmental conditions and in presence of bacterial contamination, the galvanic coupling of copper and silver in the alloy would induce a redox reaction: oxidation of bacterial cells in contact with the alloy and reduction at the metal sites, resulting in local pH raise. In the same conditions, at surface areas not occupied by bacteria cells, the reduction reaction takes place at silver sites and oxidation reaction at copper, resulting in release of copper ions.

We conclude that the copper–silver alloy is an effective antibacterial against bacterial contamination under dry conditions. The redox reaction due to the galvanic coupling of the metals in the alloy likely induce oxidation of bacterial cells, release of copper ions and local pH raise under environmental conditions. The combination of these three factors is responsible for the observed antibacterial efficacy of this alloy coating and it would ensure its properties in the intended environmental applications in healthcare settings. The understanding of the electrochemical reactivity of metals can be used to produce other combination of redox active metals, or an active system based on a galvanic couple, tailoring the choice of elements to the specific environment and application.

## 4 Experimental Section

*Materials and Surface Preparation Method*: 2B surface finish AISI 316 cold rolled stainless steel sheet (X5CrNiMo17‐12‐2) was cut into 25.4 × 25.4 mm (1 × 1 in.) size carriers.^9^, ^10^, ^15^ Carrier size of 25 × 75 mm was used for CLSM and pH monitoring measurements. The AISI 316 carriers were electroplated at a current of 4 A dm^−2^ for 1 min in a commercially modified copper–silver bath at Elplatek A/S Galvanord. Prior to the electroplating process, the specimens were cathodically degreased (3 ± 0.5 V for 2 min), rinsed in deionized water and surface activated in a Wood's nickel strike (4.5 ± 0.5 A dm^−2^ for 2 min). Copper–silver alloy‐coated and uncoated AISI 316 carriers were used as test and control carriers, respectively.

*Efficacy of Copper Alloys Surfaces as Sanitizer*: The tests were performed according to the guidelines reported in the *Test method for Efficacy of Copper Alloy Surfaces as a Sanitizer* approved by the US EPA and using Good Laboratory Practice (GLP).^9^ On the day prior to the test, five carriers per each material and organism were cleaned with 70% isopropyl alcohol, rinsed with deionized water, and allowed to air dry. After sterilization by dry heat, each carrier was placed in individual sterile plastic Petri dishes.^13^ Six stainless steel and three copper–silver‐coated carriers per organism were used for the carrier viability, carrier quantitation, neutralizer sterility, neutralizer confirmation, and carrier sterility controls according to the protocol guidelines.^9^ The test controls were performed in parallel per each test. *Staphylococcus aureus* ATCC 6538, Methicillin Resistant *Staphylococcus aureus* (MRSA) ATCC 33592, *Enterobacter aerogenes* ATCC 13048, and *Pseudomonas aeruginosa* ATCC 15442 were revived from –80 °C stock cultures, streaked on Tryptone Soy Agar (TSA) (Oxoid CM0131) and incubated for 24 h at 36 ± 1 °C (27 ± 2 °C for *E. aerogenes*). Selected colonies were transferred to 1 mL Tryptone Soy Broth (TSB) (Oxoid CM0129) incubated for 24 ± 2 h at 36 ± 1 °C (27 ± 2 °C for *E. aerogenes*). Two 10 µL loopfuls of culture were transferred to 10 mL TSB and incubated for 24 ± 2 h at 36 ± 1 °C (27 ± 2 °C for *E. aerogenes*). This step was repeated three times. 4.7 mL of the bacteria suspension was transferred to a new tube and 0.25 mL heat‐inactivated fetal bovine serum (FBS, Sigma F2442) and 0.05 mL Triton X‐100 (Sigma‐Aldrich) were added to yield 5% FSB and 0.01% Triton X‐100 organic soil load. The carriers were spread with 0.02 mL of inoculum within 1/8 in. (≈3 mm) of the edges of the carriers and allowed to dry in a sterile bench for ≈20 min. A relative humidity of 25% and a laboratory temperature of 23 ± 2 °C were recorded during the experiments. After 120 min, the carriers were transferred to individual 50 mL falcon tubes containing 20 mL of neutralizer solution (Modified Letheen broth: Letheen broth + 0.07% Lecithin + 0.5% Tween 80). The tubes were sonicated for 5 min at 28 kHz (Delta 220; Deltasonic, Meaux, France) and rotated to collect bacteria. 10^−1^ to 10^−4^ serial dilutions in phosphate buffered saline (PBS) (Oxoid BR0014G) were made and 1 mL plated in duplicates on TSA plates. The plates were placed in a sterile bench with lids ajar in order to dry before the incubation for 48 h at 36 ± 1 °C (27 ± 2 °C for *E. aerogenes*). Plates with colony numbers in the range 5–300 were used in the evaluation. CFU per carrier were calculated as average number colonies per plate at respective dilution, multiplied by the dilution factor and the volume of the neutralized solution and divided by the volume plated. The geometric mean of the number of organisms surviving on control and test carriers was reported and used for the calculation of the percentage reduction.^9^ Testing of the antimicrobial susceptibility of MRSA ATCC 33592 against oxacillin was also performed according to the EPA protocol guidelines. *Staphylococcus aureus* ATCC 25923 was used as control organism and the inhibition zone was interpreted according to the guidelines of Clinical and Laboratory Standards Institute.^27^

*Continuous Reduction of Bacterial Contamination on Copper Alloy Surfaces*: The tests were performed according to the guidelines reported in the Test method for the Continuous Reduction of Bacterial Contamination on Copper Alloy Surfaces approved by the US EPA and using Good Laboratory Practice (GLP).^10^ The test procedure was followed as outlined in the previous section and five replicates per organism per time point were used.^13^ The carriers (25 copper–silver coated test carriers, 15 stainless steel control carriers, 16 stainless steel carriers for quantitation and viability control per each organism) were inoculated with 5 µL of the inoculum at “time 0” and allowed to air dry in sterile conditions. At 2, 6, 12, 18, and 24 h after the initial inoculation, five copper–silver electroplated carriers, three stainless steel control carriers, and three stainless steel carriers for quantitation control were recovered. These carriers were inoculated one, two, four, six, and eight times, respectively. The remaining carriers were reinoculated with 5 µL of the inoculum after 3, 6, 9, 12, 15, 18, and 21 h. The recovered carriers were transferred to individual 50 mL falcon tubes containing 20 mL of neutralizer solution, sonicated for 5 min at 28 kH (Delta 220; Deltasonic, Meaux, France) and rotated to mix. Serial dilutions (10^−1^–10^−4^) were made in PBS and 1 mL plated in duplicates on TSA plates. After drying, the plates were incubated for 48 h at 36 ± 1 °C (27 ± 2 °C for *E. aerogenes*). Colony numbers in the range 5–300 were used in the calculations.

*Modified Live/Dead Staining Assay and CLSM*: A modified live/dead dye mixture containing 0.2% of SYTO 9 Green‐Fluorescent Nucleic Acid Stain (Invitrogen, USA) and 0.2% of SYTOX AADvanced Dead Cell Stain (Invitrogen, USA) in MilliQ water was used to visualize and follow‐up the killing process of bacterial films in contact with the copper–silver alloy‐coated surface. SYTO 9 can penetrate both intact (live cells) and compromised (dead cells) membranes, while SYTOX AADvanced stains only compromised cells.^28^ The modified dye mixture was designed to allow the direct inspection of bacterial cells on the copper–silver alloy‐coated substrate. Copper surfaces were found to interfere and absorb the fluorescent signal of propidium iodide, which is the commonly used dye for dead cell stain.^29^ This effect is due to the characteristic light absorption of copper surfaces and results in decrease or elimination of the observed fluorescent signal.^30^ SYTOX AADvanced was used instead since it is characterized by an emission spectrum shifted to longer wavelengths and therefore it is possible to visualize its signal in contact with copper surfaces. *Staphylococcus aureus* 8325 or *Pseudomonas aeruginosa* PAO1 were revived from –80 °C stock cultures, streaked on lysogeny broth (LB) agar plates (5 g L^−1^ yeast extract (Oxoid, Roskilde, Denmark), 10 g L^−1^ tryptone (Oxoid), 10 g L^−1^ NaCl (Merck, USA), pH 7.5) and incubated for 24 ± 2 h at 37 ± 1 °C. The modified live/dead dye mixture was applied on the inoculated plates that were incubated in dark for 5–10 min. Using a 5 µL inoculating loop, the stained bacteria were transferred from the plates to the copper–silver alloy‐coated or uncoated AISI 316 25 × 75 mm carriers, mimicking a bacterial biofilm, and covered by a glass cover slide. The inoculated carriers were immediately inspected at a Zeiss LSM 880 inverted confocal laser scanning microscope using a Plan‐Apochromat 63 × /1.40 oil differential interference contrast [DIC] objective (Zeiss, Germany). A 488 nm laser was used for excitation and a 561 nm filter for emission in order to capture both the signal from SYTO 9 (emission maxima 498 nm) and SYTOX AADvanced (emission maxima 647 nm). Bacteria at the metallic substrates were imaged as a 135 µm × 135 µm field with ≈0.5 µm increments in the *Z* direction. The stacks of images were captured every 5 min within 100 min time series.

*Image Processing and Biomass Quantification*: Image processing was done using the IMARIS software package (Bitplane AG, 451 Switzerland). Quantification of the biomass as ratio of live and dead cells was performed for three experimental repeats of each combination of test organism and material by using COMSTAT 2 (www.comstat.dk) using a threshold factor of 5 without connected volume filtering.^31^, ^32^

*pH Monitoring at the Metallic Surfaces*: *Staphylococcus aureus* 8325 was from ‐80 °C stock culture, streaked on LB plates, and incubated for 24 ± 2 h at 36 ± 1 °C. A single colony was added to 5 mL LB broth and incubated for 24 ± 2 h at 36 ± 1 °C. Bacterial cells were harvested at 4000 g for 5 min, resuspended in 0.15 m NaCl solution, and adjusted to OD_600_ 2.0 by using a spectrophotometer (UV 1800, Shimadzu, Japan). 0.15 m NaCl solution 0.5% agarose was melted and 4 mL poured in a one‐well glass slide (16 × 50 × 5 mm) with a removable well (Ibidi, Germany). The agarose was allowed to cool to room temperature and solidify for at least 10 min where after, the gel matrix was inverted in order to expose the smoother side. 250 µL of *S. aureus* 8325 bacterial suspension was spread on the surface and left to air dry for 5 min. pH measurements were done using pH microelectrodes (PH25, tip diameter ≈25 µm, Unisense A/S) with a linear range between pH 4–9, a 90% response time <10 s. The pH microelectrodes were used in combination with a reference microelectrode (REF‐100, tip diameter of ≈100 µm; Unisense A/S) immersed in the agarose matrix to ensure electrical contact to the microelectrode. The pH microelectrode was calibrated from sensor readings in three pH buffers (pH 4.01, 7.00, and 10.01, at experimental temperature) and responded linearly to pH over the calibration range with a signal to pH ratio of ≈56 mV per pH unit. The pH electrodes were connected to a multimeter (Unisense A/S) and data acquisition was done in PC running software (SensorTrace Suite; Unisense A/S). During operation, the microsensors were mounted on a PC‐interfaced motorized micromanipulator (MM33‐2, MC‐232; Unisense A/S) controlled by dedicated positioning software (SensorTrace Suite; Unisense A/S). The inoculated gel matrix was placed on copper–silver alloy‐coated or uncoated AISI 316 substrate carrier (25 × 75 mm) and the electrodes were carefully positioned at a safe distance (<100 µm) from the metallic surface as rapidly as possible. However, in one replicate, the positioning of the sensor was slow and the initial rise in pH was not recorded. Therefore, this replicate was fitted with a model (*y* = *a*(*ln*(*x*)) + *b*) that allowed extrapolation of its initial pH rise. pH was also monitored at the surface of copper–silver alloy‐coated or uncoated AISI 316 without bacterial inoculum. Here, the pH dynamics were faster than when bacteria were present so in order to capture the initial pH rise, the sensors were positioned close to the surface (<100 µm) and a drop of 0.5% low melting point agarose (Ultra Pure LMP Agarose, Invitrogen, USA) was deposited on the surface covering both the sensor and reference electrode tips. The drops (100 µL) were deposited at a temperature of 28 °C and the agarose solidified immediately upon contact with the alloy‐coated surface.

*Exposure of S. aureus* 8325 *to 1 m Tris‐HCl Buffer at pH 8.0 to 9.5*: *Staphylococcus aureus* 8325 was revived from –80 °C stock culture, streaked on Brain Heart Infusion (BHI) agar plates (Oxoid, CM1135) and incubated for 24 ± 2 h at 36 ± 1 °C. Single colonies were added to 5 mL BHI broth and incubated for 24 ± 2 h at 36 ± 1 °C. 1 m Tris–HCl buffers (121.1 g Tris Base (Trizma, Sigma‐Aldrich), 700 mL dH_2_O) were prepared and the pH was adjusted to 8.0, 8.5, 9.0, 9.5 using concentrated HCl (Sigma‐Aldrich). Bacterial suspensions were adjusted to OD_600_ 2.0 by using a spectrophotometer (Novaspec III Visible Spectrophotometer, Amersham Biosciences) and 1 mL was transferred in Eppendorf tubes (Eppendorf AG, Hamburg). Bacterial cells were harvested at 4000 g for 5 min and resuspended in 1 mL 1 m Tris–HCl buffers. Bacterial suspensions were sampled after 1 and 24 h exposure time. The density of bacterial survival in suspension (CFU mL^‐1^) was determined by serial dilution and plating on BHI‐agar. All experiments were conducted in three biological replicates; average values and standard deviation among replicates are reported (Table 2).

## Conflict of Interest

The authors declare no conflict of interest.
